# Association of integrin-β2 polymorphism and expression with the risk of rheumatoid arthritis and osteoarthritis in Egyptian patients

**DOI:** 10.1186/s12920-023-01635-3

**Published:** 2023-08-29

**Authors:** Aliaa M. Selim, Yumn A. Elsabagh, Maha M. El-Sawalhi, Nabila A. Ismail, Mahmoud A. Senousy

**Affiliations:** 1 Department of Biochemistry, Faculty of Pharmacy, Cairo University, 11562 Cairo, Egypt; 2 Department of Rheumatology and Clinical Immunology, Internal Medicine, Kasr Al- Ainy, Faculty of Medicine, Cairo University, Cairo, Egypt

**Keywords:** Integrins, ITGB2, Polymorphism, Rheumatoid arthritis, Osteoarthritis

## Abstract

**Background:**

The genetic architecture of rheumatoid arthritis (RA) and osteoarthritis (OA) are still unclear. Although RA and OA have quite different causes, they share synovial inflammation, risk factors, and some disease-associated genes, including the integrin subunit β2 (ITGB2)/CD18 gene involved in extracellular matrix interactions and immune cell signaling. However, the functional role of ITGB2 genetic variants, its circulating expression pattern, and their clinical usefulness in RA and OA remain unexplored. Our study appraised the association of ITGB2 rs2070946 single nucleotide polymorphism with the vulnerability to RA and OA and its influence on ITGB2 mRNA expression, along with the potential of serum ITGB2 expression in RA and OA diagnosis.

**Methods:**

This study included 70 RA patients, 70 primary OA patients, and 60 healthy volunteers. Genotyping and gene expression analysis were performed using qPCR. Bioinformatics analysis was employed to construct the protein-protein interaction (PPI) network of ITGB2.

**Results:**

Serum ITGB2 mRNA expression was upregulated in both RA and OA compared to healthy controls. ITGB2 rs2070946 was associated with escalating risk of both diseases. RA patients harboring the rs2070946 CC or TC + CC genotypes had higher serum ITGB2 expression than the TT genotype carriers. Likewise, OA patients having the minor homozygote CC genotype had higher serum ITGB2 expression than those carrying the TT, TC or TT + TC genotypes. Serum ITGB2 expression showed profound diagnostic potential for RA and OA in receiver-operating characteristic analysis. In RA, serum ITGB2 expression positively correlated with rheumatoid factor and disease activity score 28 (DAS28). The ITGB2-PPI network enriched in cell-cell adhesion, ICAM-3 receptor activity, T-cell activation, leukocyte adhesion, complement binding, and NF-κB, tumor necrosis factor, and interleukin signaling pathways.

**Conclusion:**

These findings embrace the impact of ITGB2 rs2070946 as a novel genetic biomarker of both RA and OA, which could alter the ITGB2 expression. Serum ITGB2 expression could aid in timely diagnosis of RA and OA.

**Supplementary Information:**

The online version contains supplementary material available at 10.1186/s12920-023-01635-3.

## Introduction

Rheumatoid arthritis (RA) and osteoarthritis (OA) are considered among the most communal forms of inflammatory arthritis. Although they differ in causes, RA and OA have many cohesions, including synovial inflammation and risk factors like age, obesity, and female gender [1]. The economic burden due to the prevalence of RA and OA is high, particularly from high cost of medical treatment, job loss as well as debility [1, 2]. This warrants the identification of disease-associated genes and related molecular mechanisms that will aid in timely diagnosis and optimal management of these conditions.

RA is a complex, chronic autoimmune disease caused by close-fitting interaction of synovial cells with innate and adaptive immune mediators, with a prevalence of 460 per 100,000 people worldwide [3, 4]. Besides, RA is elicited by several environmental, genetic, and epigenetic factors and clinically manifests as severe pain and swelling of chronic inflamed joints that result in joint devastation and function disability [5]. The current diagnosis of RA relies on the 2010 RA Classification Criteria developed by the American College of Rheumatology and European League Against Rheumatism (ACR/EULAR), which have a 60% specificity and 84% sensitivity for classification as RA [6], necessitating the identification of novel biomarkers to bolster an efficient diagnosis of RA in a timely manner.

OA is a highly predominant debilitating joint degenerative disease which affects nearly 7% of people globally [2]. OA is pictured by progressive loss of articular cartilage due to wear and tear overtime along with remodeling of the underlying bone resulting in painful, swelled, and stiffened joints. The pathogenesis of OA is a composite process that is affected by personal-related factors, joint injury or overuse as well as genetic predisposition such as cytokine genes polymorphism [7]. Interestingly, OA is a low-grade inflammatory disease affecting all joint tissues and is accompanied with dysregulated intracellular mechanisms mediated by the innate immune system [8]. The inflammatory response in OA promotes synovitis and progression of cartilage and bone destruction via the secretion of chemokines, cytokines, and other molecules, that can be detected in the synovial fluids which are enriched in macrophages, T cells, and dendritic cells; notably, DC3s, CD1c^+^, and others [9].

OA is currently diagnosed with radiography which can take years and even decades to progress. However, symptoms often arise before any radiographic abnormality [7]. Thereby, the identification of new biological markers as timely indicators of OA risk becomes necessary.

Integrins are transmembrane proteins composed of heterodimers of α and β chains that exist in nearly 24 unique non-covalently interacting combinations of 18 types of α subunits and 8 types of β subunits. Indeed, they act as receptors that establish interactions between cells as well as cell-extracellular matrix (ECM) interactions [10]. Upon ligand binding, integrins induce the expression of certain cytokines and proteases and undergo a conformational change that allow binding to the ECM [11, 12].

β2-integrins, the most circulating leukocytes integrins, are a subgroup of integrins which share a common β2 subunit, also known as CD18, and different α-subunits, including αLβ2, αMβ2, αXβ2, and αDβ2 [13]. β2-integrins have different expression and ligand binding in different leukocyte subpopulations and are essential for pro-inflammatory as well as anti-inflammatory processes. Additionally, β2-integrins are involved in different aspects of immune cell function, including immune cell recruitment, interactions, and signaling. Indeed, dysregulation of these functions is implicated in inflammation, autoimmunity, and infection [14]. Notably, the deregulation of integrin subunit β2 (ITGB2) expression in RA has been previously reported [15] and was recently identified among a nine hub genes related to the pathogenesis of RA [16]. Moreover, ITGB2 was identified by a bioinformatics study as one of the top 20 disease-associated genes common in the pathophysiology of both RA and OA, suggesting that both diseases have shared molecular mechanisms [15]. However, the expression pattern of ITGB2 and its clinical usefulness in RA and OA are still elusive.

Several single nucleotide polymorphisms (SNPs) have been estimated for their impeccable roles in autoimmune and inflammatory diseases and are implemented as genetic biomarkers [17]. Genetic variants of some α and β-integrin genes were associated in genome wide association studies (GWAS) with inflammatory and autoimmune diseases, including inflammatory bowel diseases, coronary heart diseases, and systemic lupus erythematosus [18–20]. The ITGB2 genetic variants have been associated with the risk of papillary thyroid cancer [21], Behçet’s disease [22], and coronary heart disease [23]. In particular, the ITGB2 promoter SNP rs2070946 was associated with the development of papillary thyroid cancer [21]. However, the repercussion of ITGB2 variants in predisposing RA and OA risk is not yet known.

As RA and OA seem to share some disease-associated genes, including ECM-related genes and probably genetic factors [24–26], the current study attempted to investigate the association of ITGB2 rs2070946 genetic variant with the susceptibility to RA and OA and its influence on ITGB2 mRNA expression. We also explored the potential of circulating ITGB2 expression as a diagnostic biomarker for RA and OA. The correlations between ITGB2 polymorphism and expression with the clinicopathological data of both diseases were also assessed. Additionally, bioinformatics analysis was employed to construct and analyze the molecular network of ITGB2.

## Subjects and methods

### Subjects

Two hundred Egyptian participants, aged 50–70 years old, were included in this case-control study. They were classified as 70 RA patients and 70 primary knee OA patients, together with 60 healthy participants who were sex- and age-matched to both groups. All patients were recruited from the Rheumatology and Immunology Unit of Kasr Al-Ainy Hospital, Cairo, Egypt and were recently diagnosed without prior experience to treatment.

Full history taking and clinical assessment were done for all participants. RA was diagnosed as stated by the 2010 ACR/EULAR classification criteria [6]. Disease activity score 28 (DAS28) was calculated to assess the disease activity of RA [27]. The exclusion criteria included RA patients who have malignancy or other autoimmune diseases, or who were getting treatment.

Knee OA was diagnosed according to the clinical and radiographic revised criteria of ACR (2016) [28]. Kellgren–Lawrence (KL) grading scale was used as a radiographic OA classification scale system to assess the structural features of knee OA [29].

The exclusion criteria for the primary OA group included patients who have any autoimmune or other inflammatory diseases, trauma or patients who previously received oral or intra-articular injection.

### Ethics declaration

Written informed consent was obtained from all participants before the beginning of the study. The ethics committee of the Faculty of Pharmacy, Cairo University approved the study protocol as well as the informed consent (approval number: BC2735) which followed the ethical standard of the Helsinki declaration revised in 2008.

### Sample collection

Six milliliters were collected from venous blood samples of each participant into two different vacutainer collection tubes (Greiner Bio-One, Frickenhausen, Germany). The first tube contained EDTA for whole blood collection, while the other tube was indicated for serum separation. The EDTA-blood samples were then stored at -80 °C till DNA extraction and genotyping. Within 2 h, the serum tubes were centrifuged at 4000 g for 15 min; the separated serum was then stored at -80 °C for total RNA extraction and other biochemical parameters.

### Biochemical and clinical measurements

Routine biochemical and clinical investigations as well as knees x-ray were performed. Serum C-reactive protein (CRP) and rheumatoid factor (RF) levels were measured using commercial sandwich ELISA kits (Biovision, US). Erythrocyte sedimentation rate (ESR) was measured using Westergren tubes (mm/h). Serum alanine aminotransferase (ALT) and aspartate aminotransferase (AST) colorimetric assays were routinely conducted for the evaluation of liver function of patients before receiving treatment using commercially available kits (Biodiagnostic, Egypt).

The patients’ radiographs were interpreted by a rheumatologist for the severity of knee OA using KL scale [29]. A rheumatologist also evaluated the RA patients and collected their records of clinical and demographic data and calculated the DAS28 score [27] to reflect the RA clinical disease activity.

### SNP selection

We searched for SNPs in ITGB2 gene that have positive reported results or trends. We found five SNPs (rs235326, rs2070947, rs2070946, rs684, and rs760456), which have been reported but have not yet been tested in an Egyptian population. Then, to filter these results, we used criteria of: SNP with minor allele frequency (MAF) > 10%, and has functional, regulatory feature consequences, or clinical significance in dpSNP and Ensembl (release 109, 2023) databases. rs684 has a MAF < 10% in some populations. Besides, the rs684 and rs2070947 were recorded as benign in dpSNP, rs235326 was reported as synonymous variant in Ensembl, while rs760456 has no reported functional consequence or regulatory features in either database, thus were excluded. rs2070946 was reported to be a promoter SNP (a regulatory region variant) and seems the only functionally relevant SNP, thereby was chosen for analysis.

### DNA extraction and genotyping

Two hundred microliters of EDTA-blood samples were used to extract genomic DNA using the QIAamp DNA MiniKit (Qiagen, Germany) according to the manufacturer’s instructions. The concentration and purity of the yield were measured at OD260 and OD260/OD280 ratio, respectively using Implen NanoPhotometer® P-Class 300 (Implen, Germany). The genotyping of SNP rs2070946 (T/C) was performed using real-time polymerase chain reaction (PCR) with the TaqMan allelic discrimination assay as previously reported [30]. Previously designed primer/probe [Assay ID: C_ 15868086_10, Catalog number: 4,351,379] were used. PCR was conducted on StepOne Applied Biosystems Real-Time PCR System (Thermo Fisher Scientific, USA) under the following cycling conditions: 95 °C for 10 min, followed by 40 cycles at 92 °C for 15 s and 60 °C for 90 s. Fluorescence was assessed at the end of each cycle.

### Serum ITGB2 mRNA assay using reverse transcriptase-quantitative polymerase chain reaction (RT-qPCR)

Total RNA was extracted from 200 µl serum by miRNeasy Mini kit (Qiagen, Germany) with the supplied QIAzol lysis reagent according to the manufacturer’s recommendations. The concentration and purity of the resulting RNA were evaluated by measuring the OD260 and OD260/OD280 ratio with Implen NanoPhotometer® P-Class 300 (Implen, Germany). The extracted RNA samples were then converted to complementary DNA (cDNA) using High-Capacity cDNA Reverse Transcription kit (Applied Biosystems, Thermo Fisher Scientific, San Jose, USA) as written in the kit’s instructions. Reverse transcription was attempted on 0.1 µg of total RNA in a final volume 20 µL RT reactions. The following thermal cycler conditions were implemented: 10 min at 25 °C, 120 min at 37 °C, and 5 min at 85 °C. The cDNA was stored at – 20 °C until further analysis.

The quantitative detection of ITGB2 gene expression was performed on StepOne Applied Biosystems Real-Time PCR System (Thermo Fisher Scientific, San Jose, USA) using Maxima SYBER Green/ROX qPCR Master Mix (Thermo Fisher Scientific, San Jose, USA) and customized primers according to the instructions provided by the manufacturer by applying the following cycling conditions: 95 °C for 10 min followed by 40 cycles of 15 s at 95 °C and 60 s at 60 °C. We used human glyceraldehyde 3-phosphate dehydrogenase (GAPDH) as the housekeeping reference gene. The specificity of primers to target genes was checked using NCBI primer Blast and the primers were custom-made by Invitrogen (Carlsbad, USA). The following were the primers’ sequences used in the study: ITGB2-forward 5′-TGCGTCCTCTCTCAGGAGTG-3′, ITGB2-reverse 5′-GGTCCATGATGTCGTCAGCC-3′, GAPDH-forward 5′-GGCCCTGACAACTCTT-TTCATC-3′, and GAPDH-reverse 5′-CTGGTTGAGCACAGGGTACT-3′.

The specificity of PCR products was confirmed by melting curve analysis. The ΔΔCt method was used for calculating the changes in target gene expression and these changes were presented as fold change using the formula 2^−ΔΔCt^ [31].

### Functional analysis

Bioinformatics analysis was carried out to construct the protein–protein interaction (PPI) network of ITGB2 using the STRING database version 11.5 (https://string-db.org/). The STRING database was also used to carry out the functional enrichment analysis in particular the gene ontology (GO) biological process and molecular function, KEGG pathways, and reactome of the PPI.

### Statistical analysis

We used GraphPad Prism 6.0 (GraphPad Software, CA) to perform statistical analyses. Values are expressed as number (percentage), mean ± standard deviation (SD) or median (interquartile range) (IQR) when suitable. Fisher exact or χ2 tests were performed to compare categorical data. One-way ANOVA followed by the Tukey post-hoc test or Kruskal-Wallis test followed by Dunn’s multiple comparisons test were used to compare continuous variables when proper. Odds ratio (OR) and 95% confidence interval (CI) were computed from logistic regression analyses to evaluate the associations between the SNP genotypes and the risk of RA and OA or disease activity using age and sex as cofounders. Bonferroni correction was used to correct the *P*-values for multiple testing (5 genetic models) in the SNP data analysis. In this study, we conducted the receiver-operating characteristic (ROC) curve analysis and calculated the area under the curve (AUC) in order to estimate the discriminative value of ITGB2 expression level. We considered AUC = 0.6 to 0.69 as significant discriminator, AUC = 0.7–0.89 as potential discriminator and AUC = 0.9 or higher as excellent discriminator. Correlations between ITGB2 expression and laboratory and clinical parameters were computed using Spearman correlation. For all tests, *P* < 0.05 was considered to be statistically significant.

The sample size was calculated using G-Power software version 3.1.9.7. A minimum total sample size of 200 yielded a one-tailed power of 0.8 at significance level α = 0.05, with assumed odds ratio of 2.5 and case/control ratio = 1 using the Fischer exact test.

## Results

### Demographic, biochemical, and clinicopathological characteristics of RA and OA patients

Table 1 displays the demographic, laboratory, and clinical data of the study groups. RA patients had higher CRP, ESR, RF, and total leucocyte count (TLC) than healthy controls and OA patients (*P* < 0.0001 for each). Levels of CRP, ESR, RF, and TLC in OA patients were comparable to those in healthy controls (*P* > 0.05). Both RA and OA patients had normal liver function. Concerning the clinicopathological data, 45.72% and 54.28% of RA patients had a mild/moderate and severe DAS28, respectively. Likewise, 45.72% and 54.28% of OA patients had KL score ≤ 2 and > 2, respectively.

**Table 1:** Demographic, biochemical, and clinicopathological data of RA and OA patients

Parameter	Healthy controls (n = 60)	RA (n = 70)	OA (n = 70)	*P-*value
**Age (years)**	55.11 ± 4.70	53.19 ± 5.2	56.40^b^ ± 5.49	**0.001**
**Sex, n (%)**				
**Female** **Male**	54 (90) 6 (10)	68 (97.2) 2 (2.8)	67 (95.7) 3 (4.3)	0.47
**CRP, mg/L**	6 (3.95-7)	34^a^ (22.50–55)	5^b^ (4-7.5)	**< 0.0001**
**ESR (mm/h)**	13.08 ± 0.62	44.58^a^ ± 3.39	12.95^b^ ± 0.75	**< 0.0001**
**RF**	5.722 ± 0.25	41.23^a^ ± 5.14	5.506^b^ ± 0.23	**< 0.0001**
**ALT, U/L**	22.17 ± 1.26	22.28 ± 0.89	22.36 ± 1.097	0.608
**AST, U/L**	22.17 ± 1.35	22.54 ± 1.08	22.58 ± 1.20	0.11
**TLC (x 1000/mm** ^**3**^ **)**	6.26 ± 0.23	8.04^a^ ± 0.55	6.40^b^ ± 0.23	**< 0.0001**
**DAS28** **Mild-Moderate, n (%)** **Severe, n (%)**	-	5.011 ± 0.98 32 (45.72) 38 (54.28)	-	-
**KL score** **≤ 2, n (%)** **> 2, n (%)**	-	-	2.77 ± 0.8 32 (45.72) 38 (54.28)	-

Values are expressed as mean ± SD or number (percentage). CRP levels were not normally distributed and are expressed as median (interquartile range). *P*-values of one-way ANOVA followed by Tukey post-hoc test, Kruskal-Wallis test followed by Dunn’s multiple comparison, or chi square tests are presented. Multiplicity adjusted *P*-values following Tukey multiple comparison test was computed using GraphPad Prism 6.0. ^a^ significant difference from control group; ^b^ significant difference from RA group. Bold indicates statistical significance, *P* < 0.05. ALT, alanine aminotransferase; AST, aspartate aminotransferase; CRP, C-reactive protein; DAS28, disease activity score 28; ESR, erythrocyte sedimentation rate; KL, Kellgren–Lawrence; OA, osteoarthritis; RA, rheumatoid arthritis; RF, rheumatoid factor; TLC, total leukocyte count.

### Association of rs2070946 (T/C) with the risk of RA

SNP genotyping was performed while coding the subjects’ case-control status. The MAF of rs2070946 in the healthy controls (C = 0.39) was close to the highest population MAF (C = 0.35) mentioned in the Ensembl database (Ensembl Release 109, 2023). The distribution of rs2070946 genotypes in healthy controls, RA, and OA patients followed the Hardy-Weinberg equilibrium (*P* = 0.11, 0.7 and 0.23, respectively).

Table 2 shows the allele and genotype frequencies of ITGB2 rs2070946 in RA patients. The rs2070946 major T and minor C allele frequencies achieved a substantial difference between the patients and controls, where the minor C allele was a significant risk factor for RA [C vs. T, adjusted OR (95%CI) = 3.883 (2.334 to 6.445), *P* = 0.0005]. The rs2070946 CC genotypes were associated with an escalating RA risk in the codominant [CC vs. TT, adjusted OR (95%CI) = 22.17 (5.902 to 85.84), *P* = 0.0005]. The combined TC + CC genotype was also accompanied by high risk of RA in the dominant model [TC + CC vs. TT, adjusted OR (95% CI) = 6.024 (2.031 to 15.48), *P* = 0.0025]. Additionally, when testing the recessive model, the minor homozygote CC genotype was associated with a profound risk of RA [CC vs. TT + TC, adjusted OR (95% CI) = 9 (3.338 to 22.75), *P* = 0.0005]. Age and sex were added as confounders in the logistic regression model. The *P*-values were adjusted using Bonferroni correction. Even when applying a stringent significance level at *P* < 0.01, the results are still significant in the codominant (CC vs. TT), dominant, recessive, and allelic models.

**Table 2:** Genotype and allele frequencies of ITGB2 rs2070946 SNP (T/C) in RA patients and healthy controls

Genetic model	rs2070946 Genotype/ allele	Frequency, n (%)		Adjusted OR (95%CI)	*P*^*a,b*^ value
		Healthy controls (n = 60)	RA (n = 70)		
**Codominant**	TT	19 (31.7)	5 (7.1)	1	
	TC	35 (58.3)	30 (42.9)	3.257 (1.114–8.645)	0.245
	CC	6 (10)	35 (50)	22.17 (5.902–85.84)	**0.0005**
**Dominant**	TT	19 (31.7)	5 (7.1)	1	
	TC + CC	41 (68.3)	65 (92.9)	6.024 (2.031–15.48)	**0.0025**
**Recessive**	TT + TC	54 (90)	35 (50)	1	
	CC	6 (10)	35 (50)	9 (3.338–22.75)	**0.0005**
**Overdominant**	TT + CC	25 (41.7)	40 (57.1)	1	
	TC	35 (58.3)	30 (42.9)	0.5357 (0.261–1.063)	0.565
**Allelic**	T	73 (0.61)	40 (0.29)	1	
	C	47 (0.39)	100 (0.71)	3.883 (2.334–6.445)	**0.0005**

Values are expressed as number (percentage). ^a^adjusted by age and sex in a logistic regression model. ^b^Bonferroni correction for multiple testing was applied. Bold indicates statistical significance, *P* < 0.05. CI, confidence interval; OR, odds ratio; RA, rheumatoid arthritis.

### Association of rs2070946 (T/C) with the risk of OA

As shown in Table 3, the distribution of rs2070946 alleles in OA patients revealed a similar pattern to that in RA patients. In the codominant model, holders of the heterozygote TC and the minor homozygote CC genotypes carried a high risk of OA [TC vs. TT, adjusted OR (95% CI) = 5.971 (1.604 to 20.17), *P* = 0.025, CC vs. TT, adjusted OR (95% CI) = 35.89 (7.485 to 130.1), *P* = 0.0005]. Combined TC + CC genotype was associated with substantial OA risk in the dominant model [TC + CC vs. TT, adjusted OR (95% CI) = 10.35 (3.004 to 34.19), *P* = 0.0005]. Additionally, the minor homozygote CC genotype was a marked risk factor for OA while testing the recessive model [CC vs. TT + TC, adjusted OR (95%CI) = 8.5 (3.15 to 21.5), *P* = 0.0005]. The logistic regression analysis was controlled by age and sex as confounders. Bonferroni correction was used to correct the *P*-values for multiple testing. Similar to RA, the association is still significant when assuming a stringent significance level at *P* < 0.01.

**Table 3:** Genotype and allele frequencies of ITGB2 rs2070946 SNP (T/C) in OA patients and healthy controls

Genetic model	rs2070946 Genotype/ allele	Frequency, n (%)		Adjusted OR (95%CI)	*P*^*a,b*^*-*value
		Healthy controls (n = 60)	OA (n = 70)		
**Codominant**	TT	19 (31.7)	3 (4.3)	1	
	TC	35 (58.3)	33 (47.1)	5.971 (1.604–20.17)	**0.025**
	CC	6 (10)	34 (48.6)	35.89 (7.485–130.1)	**0.0005**
**Dominant**	TT	19 (31.7)	3 (4.3)	1	
	TC + CC	41 (68.3)	67 (95.7)	10.35 (3.004–34.19)	**0.0005**
**Recessive**	TT + TC	54 (90)	36 (51.4)	1	
	CC	6 (10)	34 (48.6)	8.5 (3.15–21.5)	**0.0005**
**Overdominant**	TT + CC	25 (41.7)	37 (52.9)	1	
	TC	35 (58.3)	33 (47.1)	0.637 (0.313–1.262)	> 0.999
**Allelic**	T	73 (0.61)	39 (0.28)	1	
	C	47 (0.39)	101 (0.72)	4.022 (2.41–6.708)	**0.0005**

Values are expressed as number (percentage). ^a^adjusted by age and sex in a logistic regression model. ^b^Bonferroni correction for multiple testing was applied. Bold indicates statistical significance, *P* < 0.05. CI, confidence interval; OA, osteoarthritis. OR, odds ratio.

### Serum ITGB2 gene expression in RA and OA patients

**Fig. 1:**
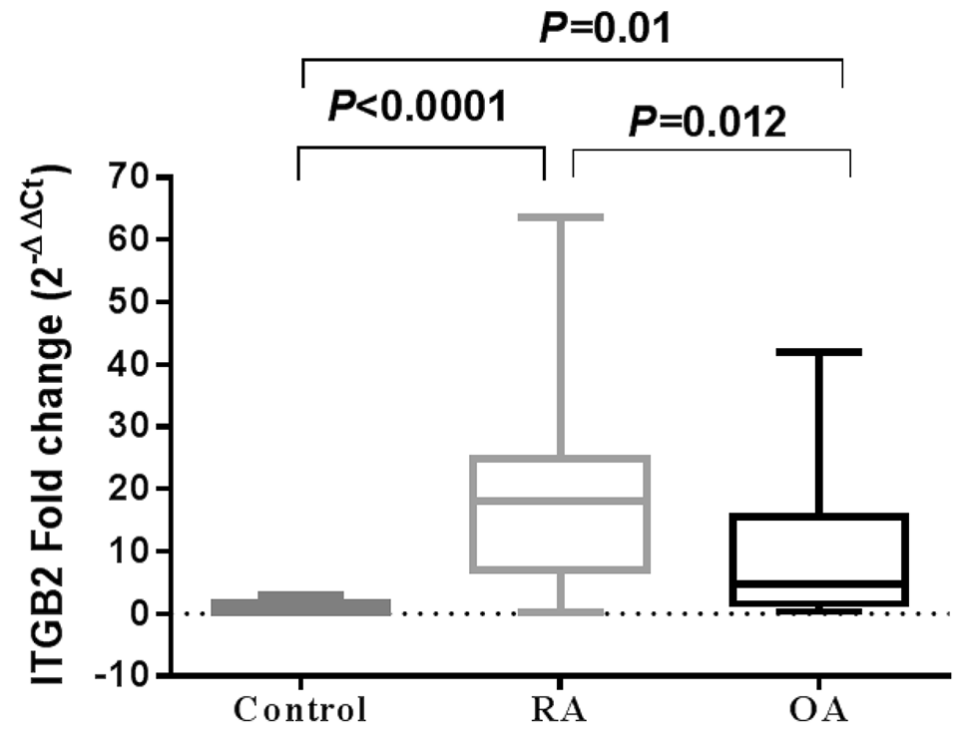
Serum ITGB2 mRNA expression in RA and OA patients. Data are expressed as box plot; the box represents the 25–75% percentiles; the line inside the box represents the median and the upper and lower lines representing the 10–90% percentiles. Data were analyzed using Kruskal-Wallis test followed by Dunn’s multiple comparison test. *P* < 0.05 indicates statistical significance

As depicted from Fig. 1, a significant upregulation in serum ITGB2 mRNA expression was recorded in RA and OA patients in comparison with healthy controls with a median (IQR) fold change of 18.04 (6.979–24.99), *P* < 0.0001 and 4.8 (1.641–15.56), *P* = 0.01, respectively. Besides, serum ITGB2 mRNA expression levels were significantly lower in patients of OA than those in RA patients, *P* = 0.012.

### Association of ITGB2 rs2070946 SNP with serum ITGB2 expression levels in RA and OA patients

**Fig. 2:**
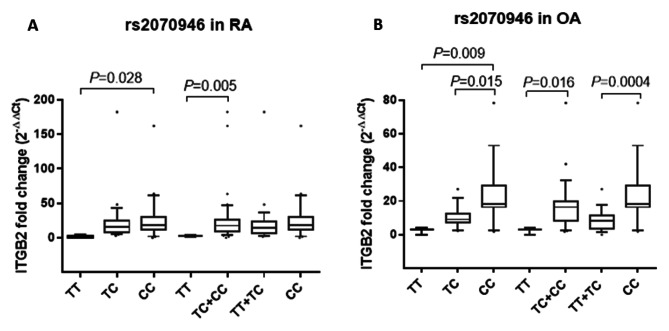
Influence of rs2070946 SNP on serum ITGB2 mRNA expression in RA and OA patients. Data are expressed as box plot; the box represents the 25–75% percentiles; the line inside the box represents the median and the upper and lower lines representing the 10–90% percentiles. Data were analyzed using Kruskal-Wallis test followed by Dunn’s multiple comparison test. *P* < 0.05 indicates statistical significance

Serum ITGB2 gene expression was assessed in RA and OA patients with the different rs2070946 genotypes to identify the impact of rs2070946 SNP on ITGB2 mRNA expression. The significant genetic models in the risk stratification (codominant, dominant, and recessive) were applied in the comparison. A substantially higher expression levels of ITGB2 mRNA were recorded in sera of RA patients carrying the CC (*P* = 0.028) or TC + CC (*P* = 0.005) genotypes than those carrying the TT genotype (Fig. 2A).

Similarly, serum ITGB2 mRNA expression levels were considerably higher in OA patients having the CC genotype than those carrying the TT (*P* = 0.009), TC (*P* = 0.015) or TT + TC (*P* = 0.0004) genotypes. Additionally, ITGB2 gene expression levels were found to be significantly higher in sera of OA patients with the TC + CC genotype than those holding the TT genotype (*P* = 0.016) (Fig. 2B).

### Association of rs2070946 SNP with laboratory and clinical characteristics in RA and OA patients

**Fig. 3:**
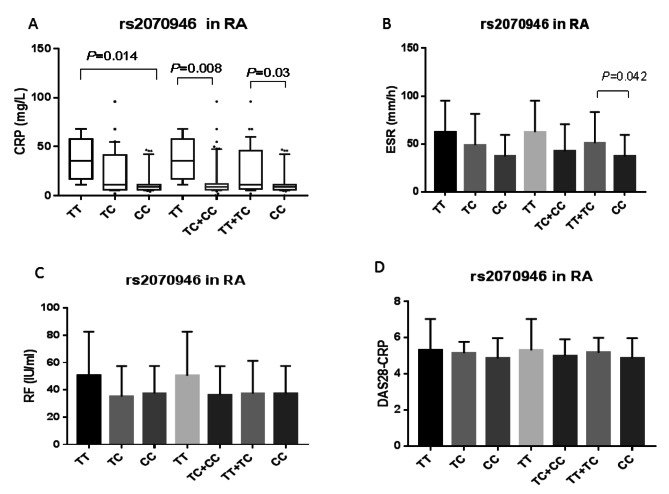
Association of ITGB2 rs2070946 with laboratory and clinical data in RA patients. Data are expressed as mean ± SD or box plot for CRP. In the box plot, the box represents the 25–75% percentiles; the line inside the box represents the median and the upper and lower lines representing the 10–90% percentiles. Data were analyzed using ANOVA followed by Tukey’s multiple comparison test for RF, ESR, and DAS28. For CRP data, the non-parametric Kruskal-Wallis test followed by Dunn’s multiple comparison test was used. Statistical significance was set at *P <* 0.05

In RA patients, serum CRP levels were markedly lower in the rs2070946 CC genotype carriers than the TT (*P* = 0.014) or TT + TC (*P* = 0.03) genotype carriers in the codominant and recessive models, respectively. Likewise, in the dominant model, serum levels of CRP were substantially lower in the TC + CC genotype carriers than those holding the TT genotype (*P* = 0.008) (Fig. 3A). Additionally, ESR levels were significantly lower in the CC genotype holders than those harboring the TT + TC in the recessive model (*P* = 0.042) (Fig. 3B). The rs2070946 SNP was not correlated with RF levels (Fig. 3C) or DAS28 scores (Fig. 3D) in RA patients. In a sub-analysis, the rs2070946 also revealed no correlation with DAS28 scores when patients were classified into mild-moderate or high disease activity (Supplementary Table S1).

**Fig. 4:**
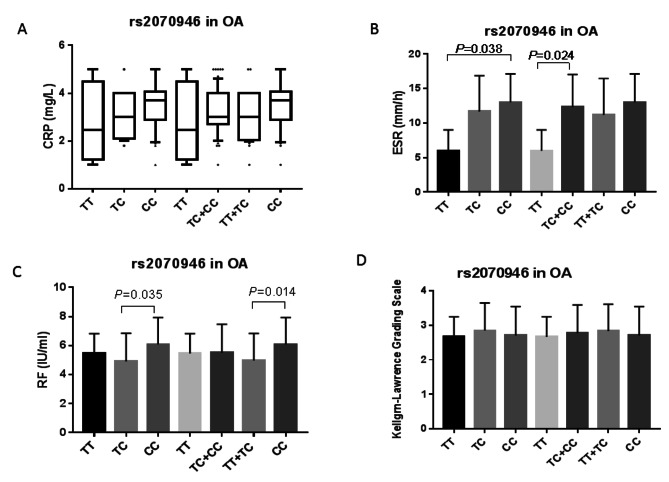
Association of ITGB2 rs2070946 with laboratory and clinical data in OA patients. Data are expressed as mean ± SD or box plot for CRP. In the box plot, the box represents the 25–75% percentiles; the line inside the box represents the median and the upper and lower lines representing the 10–90% percentiles. Data were analyzed using ANOVA followed by Tukey’s multiple comparison test for RF, ESR, and KL score. For CRP data, the non-parametric Kruskal-Wallis test followed by Dunn’s multiple comparison test was used. Statistical significance was set at *P <* 0.05

In OA patients, rs2070946 was not significantly correlated with serum CRP levels (Fig. 4A). However, significant higher ESR levels were observed in the CC genotype holders than those holding the TT genotype in the codominant model (*P* = 0.038), while the TT genotype carriers had significantly lower ESR levels compared with levels in the TC + CC genotype carriers (*P* = 0.024) (Fig. 4B). Moreover, RF levels were markedly higher in the rs2070946 CC genotype carriers than in the TC (*P* = 0.035) or TT + TC genotype (*P* = 0.014) carriers in the codominant and recessive model, respectively (Fig. 4C). The rs2070946 SNP was not correlated with KL scores (Fig. 4D) in OA patients. In a sub-analysis, we failed to find such correlation between the rs2070946 and KL scale when patients were classified into KL **≤** 2 or > 2 (Supplementary Table S2).

### Correlation of ITGB2 mRNA expression with laboratory and clinical characteristics in RA and OA patients

Intriguingly, serum ITGB2 mRNA expression levels were positively correlated with RF levels (r = 0.327, *P* = 0.03) and disease activity score (DAS28) (r = 0.312, *P* = 0.042) in RA patients. However, ITGB2 expression was not correlated with the studied biochemical and clinical parameters in OA patients (Table 4).

**Table 4:** Correlation of ITGB2 mRNA expression with laboratory and clinical data in RA and OA patients

	ITGB2	
	RA	OA
**RF**	**r = 0.327** ***P*** **= 0.030**	r = − 0.018 *P* = 0.920
**CRP**	r = 0.001 *P* = 0.994	r = − 0.103 *P* = 0.558
**ALT**	r = − 0.057 *P* = 0.71	r = − 0.132 *P* = 0.45
**AST**	r = − 0.159 *P* = 0.30	R = − 0.317 *P* = 0.064
**TLC**	r = 0.130 *P* = 0.399	r = − 0.093 *P* = 0.593
**ESR**	r = 0.0001 *P* = 1	r = − 0.197 *P* = 0.256
**Age**	r = 0.135 *P* = 0.382	r = 0.112 *P* = 0.522
**DAS28**	**r = 0.312** ***P*** **= 0.042**	-----
**KL**	-----	r = − 0.202 *P* = 0.245

Spearman rho coefficient was used. Bold indicates statistical significance, *P* < 0.05. ALT, alanine aminotransferase; AST, aspartate aminotransferase; CRP, C-reactive protein; DAS28, disease activity score 28; ESR, erythrocyte sedimentation rate; ITGB2, integrin subunit β2; KL, Kellgren–Lawrence; OA, osteoarthritis; RA, rheumatoid arthritis; RF, rheumatoid factor; TLC, total leukocyte count

### Diagnostic performance of ITGB2 mRNA expression in RA and OA patients

**Fig. 5:**
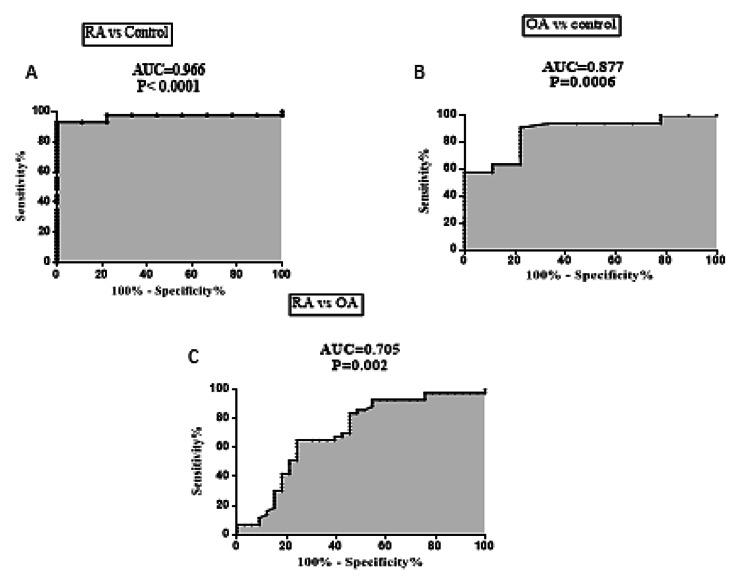
Diagnostic performance of serum ITGB2 mRNA expression levels in RA and OA using ROC curve analysis. Statistical significance was set at *P <* 0.05

ROC analysis displayed that serum ITGB2 gene expression was an excellent discriminator of RA patients from healthy controls with an AUC = 0.966, 95% CI = 0.9172 to 1.016, *P* < 0.0001, with a sensitivity of 93.02% and a specificity of 100% at a cutoff > 3.147-fold (Fig. 5A). Likewise, serum ITGB2 mRNA levels were a potential discriminator of OA patients from healthy controls with AUC = 0.877, 95% CI = 0.7571 to 0.9971, *P* = 0.0006, with a sensitivity of 57.58% and a specificity of 100% at a cutoff > 3.124-fold (Fig. 5B).

Besides, serum ITGB2 mRNA expression levels significantly discriminated RA patients from OA patients with an AUC = 0.705, 95% CI = 0.5812 to 0.829, *P =* 0.002, with a sensitivity of 93.02% and a specificity of 42.42% at a cutoff > 2.901-fold (Fig. 5C).

### Results of functional analysis

**Fig. 6:**
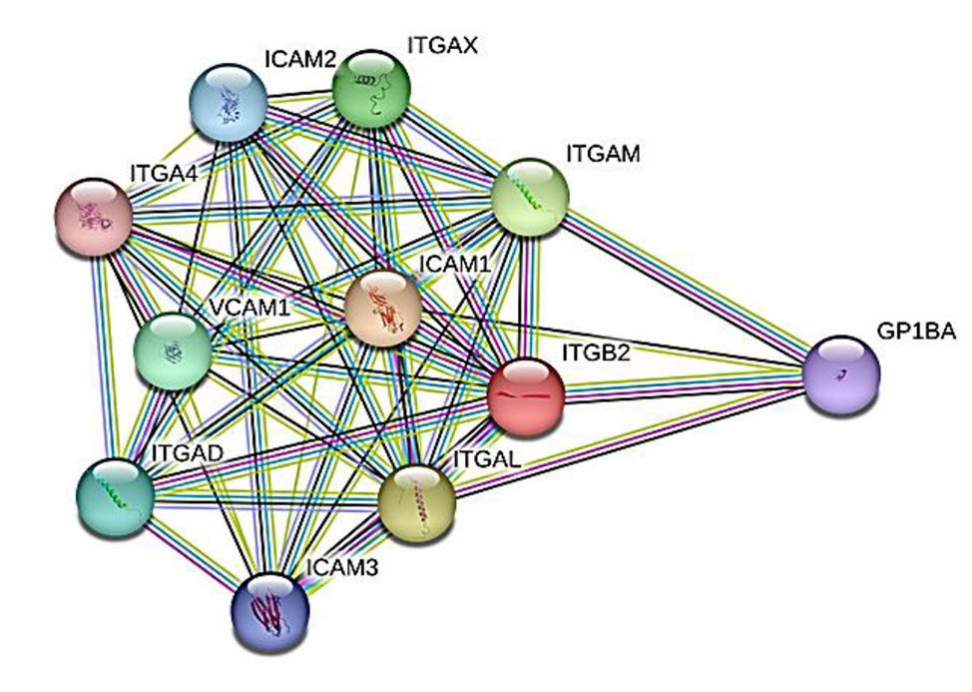
PPI network of ITGB2. The PPI enrichment *P*-value = 9.44E-15. STRING database was used. GP1BA, platelet glycoprotein Ib alpha chain; ICAM, intercellular adhesion molecule; ITGA4, integrin subunit α4; ITGAD, integrin subunit αD; ITGAL, integrin subunit αL, ITGAM; integrin subunit αM; ITGAX, integrin subunit αX; ITGB2, integrin subunit β2; PPI, protein-protein interaction; VCAM1, vascular cell adhesion molecule 1

**Fig. 7:**
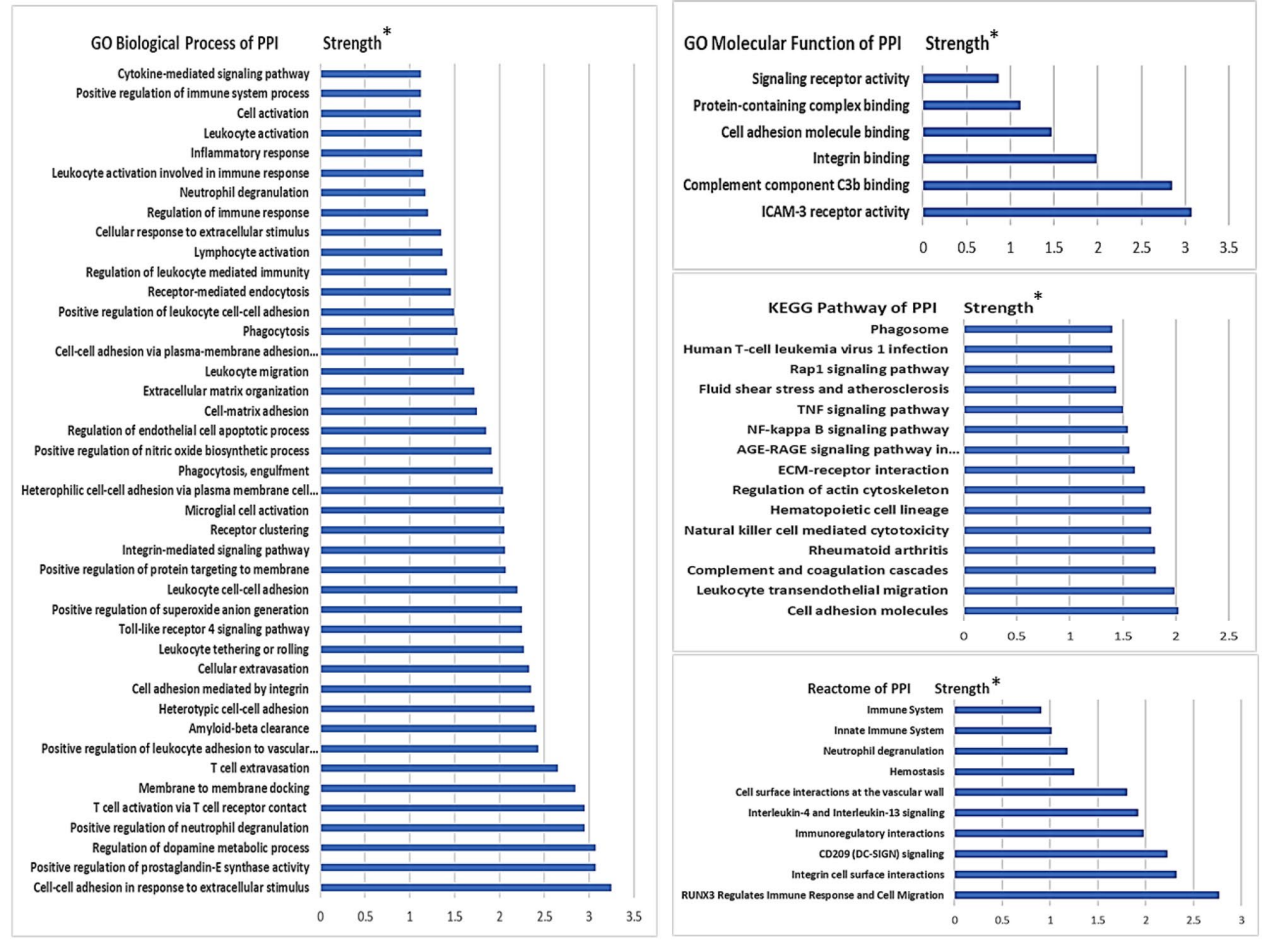
Functional enrichment analysis of ITGB2 PPI network using GO biological process, molecular function, KEGG pathways, and reactome of the PPI. STRING database was used. KEGG pathways were adopted from www.kegg.jp/kegg/kegg1.html. * false discovery rate (FDR) < 0.05. GO, gene ontology

The PPI network of ITGB2 is visualized in Fig. 6 and results of the functional enrichment analysis, including of the GO biological process, molecular function, KEGG pathways, and reactome of the PPI are configured in Fig. 7. The PPI enrichment *P*-value is 9.44E-15. The PPI consisted of 10 proteins, including intercellular adhesion molecules 1, 2, and 3 (ICAM 1,2,3), vascular cell adhesion molecule 1 (VCAM1), integrin subunits α4 (ITGA4), αD (ITGAD), αL (ITGAL), αM (ITGAM), and αX (ITGAX), and platelet glycoprotein Ib alpha chain (GP1BA, also known as CD42b). The PPI was enriched in cell-cell adhesion, ICAM-3 receptor activity, T-cell activation, leukocyte adhesion and extravasation, and complement binding as well as nuclear factor-κB (NF-κB), tumor necrosis factor (TNF), and interleukin signaling pathways.

## Discussion

The genetic architecture of both RA and OA remains unclear. Here, we are the first to reveal the association of ITGB2 or CD18 rs2070946 genetic variant with the vulnerability to RA and OA in Egyptian patients and to contemplate its genetic repercussion on ITGB2 mRNA expression. This study also highlights that serum ITGB2 expression was discriminately expressed in RA and OA patients and showed sufficient diagnostic accuracy, delineating it as a surrogate circulating biomarker of RA and OA. Interestingly, serum ITGB2 expression was also correlated with RF and disease activity in RA patients, linking it with the disease pathology.

The current study demonstrated a substantial increase in serum expression levels of ITGB2 in RA and OA patients. Similarly, ITGB2 was found to be upregulated in RA and was potentially identified as a disease-associated gene in this disease [15]. In addition, ITGB2 was highly expressed in synovial tissues and had the highest diagnostic accuracy for RA [16]. Considering OA, our finding came in accordance with a previous study which revealed an increase in ITGB2 gene expression levels in a rat model of shift–induced osteoarthritis-like changes at temporomandibular joint [32]. Furthermore, OA meniscal cells of cartilage exhibited a marked increase in ITGB2 gene expression levels than normal ones [23] and the same was observed in articular tissues of OA patients [33]. In addition, ITGB2 was uncovered as a disease-associated gene orchestrating in OA pathogenesis via bioinformatics analysis [33]. Together, these results suggest the potential of ITGB2 expression profile as a biomarker of RA and OA. In particular, we attested the relevance of ITGB2 in the clinical setting of both diseases via ROC analysis.

Regulation of integrins is achieved via extracelluar and intracellular signals; the extracellular regulation is through binding of a component to the ECM resulting in integrins activation. On the other hand, the inside-out regulation could be initiated via several mechanisms, among them comes the transcriptional upregulation [21, 34].

In this study, we emphasized the role of ITGB2 through construction of its PPI network which involved ICAMs, VCAM1, and several ITGA subunits and enriched in cell adhesion, immune and inflammatory signaling pathways, thus relating ITGB2 to the pathogenesis of RA and OA. Indeed, ITGB2 was involved in inflammatory pathways and proinflammatory cytokines [16] and deletion of its gene has been shown to result in malfunctioning leukocyte adhesion [35]. Besides, chronic inflammation was found to be regulated by CD11/CD18 in RA and spondyloarthritis patients [36]. This might explain the positive correlation between ITGB2 expression and RA disease activity, and suggest ITGB2 as a therapeutic target.

The current results displayed noticeable association of the ITGB2 promoter SNP rs2070946 with disease risk in both RA and OA, where the minor C allele was the risk allele in both diseases. Such results might indicate some sort of similarity between the genetic factors predisposing to RA and OA diseases. Likewise, this SNP has been documented to be accompanied with the development of papillary thyroid cancer, whereas another ITGB2 genetic variant (rs2070947) was not associated with this disease [21]. Notably, rs2070946 and other CD18 SNPs, including rs235326, rs760456, and rs684 also have been shown to be associated with Behçet’s disease in Koreans [22]. On the other hand, the ITGB2 SNP rs2070947 has been reported to show a significant difference in the recessive model among coronary heart disease patients in Chinese Han population [37].

We have selected the currently investigated ITGB2 SNP (rs2070946) as it is located in the promoter region of ITGB2 gene, subsequently the gene expression levels of ITGB2 might be affected. Our study assured such relation as it showed marked association between SNP rs2070946 and serum ITGB2 mRNA expression levels in both RA and OA patients. Peculiarly, harboring the minor C risk allele was associated with higher ITGB2 mRNA expression, which might magnify the disease pathology through mediating inflammatory and immune signaling. Indeed, genetic polymorphisms and subsequent changes in the secondary or tertiary structure of ITGB2 mRNA as well as its protein and their ligand-binding resulting in alteration of normal inflammatory responses and immune functions [22]. The current study revealed marked association between rs2070946 with markers of inflammation (CRP and ESR) in RA patients and surprisingly with RF and ESR in OA patients. Here, the minor C allele was associated with higher RF and ESR in OA patients, while with lower CRP and ESR levels in RA patients. This paradox warrants further investigation.

We report several limitations to this study. First, we have collected blood samples from one hospital, so selection bias might have been occurred. Second, the sample size is considered moderate, so we urge further studies on a larger sample size. Third, one population was studied, which may limit the general application of our data. Nevertheless, the present data point to the shared genetics and support the notion of common disease-associated genes between RA and OA that may aid in their timely diagnosis and optimal management.

## Conclusion

Our study is the first to embrace the ITGB2 SNP rs2070946 as a novel genetic biomarker of RA and OA and to report a marked correlation between the C allele and the risk of both diseases that could potentially impact the ITGB2 gene expression. Our results also portray serum ITGB2 gene expression as a potential diagnostic biomarker of RA and OA that might be useful in the clinical situation. These data may afford opportunities for biomarker development and novel intuitions into the shared genes of these two diseases and could pave the way for novel therapeutics targeting ITGB2.

### Electronic supplementary material

Below is the link to the electronic supplementary material.

Supplementary Material 1

## Data Availability

All data generated or analysed during this study are included in this published article and its supplementary information files.

## List of abbreviations

ALT: Alanine aminotransferase
ACR/EULAR: American College of Rheumatology and European League Against Rheumatism
AUC: Area under the curve
AST: Aspartate aminotransferase
CI: Confidence interval
CRP: C-reactive protein
DAS28: Disease activity score 28
ESR: Erythrocyte sedimentation rate
ECM: Extracellular matrix
FDR: False discovery rate
GAPDH: Glyceraldehyde 3-phosphate dehydrogenase
GO: Gene ontology
GP1BA: Platelet glycoprotein Ib alpha chain
ICAM: Intercellular adhesion molecule
IQR: Interquartile range
ITGA4: Integrin subunit α4
ITGAD: Integrin subunit αD
ITGAL: Integrin subunit αL
ITGAM: Integrin subunit αM
ITGAX: Integrin subunit αX
ITGB2: Integrin subunit β2
KL: Kellgren–Lawrence
MAF: Minor allele frequency
NF-κB: Nuclear factor-κB
OA: Osteoarthritis
OR: Odds ratio
PCR: Polymerase chain reaction
PPI: Protein-protein interaction
RA: Rheumatoid arthritis
RF: Rheumatoid factor
ROC: Receiver-operating characteristic
SNP: Single nucleotide polymorphism
SD: Standard deviation
TLC: Total leucocyte count
TNF: Tumor necrosis factor
VCAM1: Vascular cell adhesion molecule 1
