# Supplementary material for: Association of integrin-β2 polymorphism and expression with the risk of rheumatoid arthritis and osteoarthritis in Egyptian patients
Source: BMC Med Genomics. 2023 Aug 29;16:204. doi: 10.1186/s12920-023-01635-3 (PMC10463674; doi:10.1186/s12920-023-01635-3)
Supplement: Supplementary file 1 — Supplementary Material 1 [file 12920_2023_1635_MOESM1_ESM.docx]

**Supplementary Information**

**Supplementary Table S1: ITGB2 rs2070946** **SNP correlation with DAS28 scores in RA patients.**

| ***P^a^*-value** | **OR (95%CI)** | **Severe disease activity (DAS28-CRP > 5.1)**  **n=38** | **Low-Moderate disease activity**  **(DAS28-CRP ≤ 5.1)**  **n=32** | **rs2070946**  **Genotype/**  **allele** | **Genetic model** |
| --- | --- | --- | --- | --- | --- |
|  | 1 | 3 (7.9%) | 2 (6.2%) | TT | **Codominant** |
| >0.999 | 0.87 (0.139-4.803) | 17 (44.7%) | 13 (40.6%) | TC |  |
| >0.999 | 0.706 (0.115-3.837) | 18 (47.4%) | 17 (53.2%) | CC |  |
|  | 1 | 3 (7.9%) | 2 (6.2%) | TT | **Dominant** |
| >0.999 | 0.78 (0.132-4.028) | 65 (92.1%) | 30 (93.8%) | TC+CC |  |
|  | 1 | 20 (52.6%) | 15 (46.8%) | TT+TC | **Recessive** |
| 0.81 | 0.79 (0.309-1.994) | 18 (47.4%) | 17 (53.2%) | CC |  |
|  | 1 | 21 (53.3%) | 19 (59.4%) | TT+CC | **Overdominant** |
| 0.81 | 1.18 (0.463-3.128) | 17 (44.7%) | 13 (40.6%) | TC |  |
|  | 1 | 23 (0.3) | 17 (0.27) | T | **Allelic** |
| 0.708 | 0.83 (0.396-1.692) | 53 (0.7) | 47 ( 0.73) | C |  |

Values are expressed as number (percentage). ^a^adjusted by age and sex in a logistic regression model. Statistical significance was set as *P*<0.05.; CI, confidence interval; CRP, C-reactive protein; DAS28, disease activity score 28; KL, Kellgren–Lawrence. OR, odds ratio.

**Supplementary Table S2: ITGB2 rs2070946 SNP correlation with KL scores in OA patients.**

| ***P*^a^- value** | **OR (95%CI)** | **KL>2**  **n=38** | **K****L≤ 2**  **n=32** | **rs2070946**  **Genotype/**  **allele** | **Genetic model** |
| --- | --- | --- | --- | --- | --- |
|  | 1 | 2 (5.26%) | 1(3.1%) | TT | **Codominant** |
| >0.999 | 0.77 (0.049 to 7.185) | 20 (53.63%) | 13 (40.6%) | TC |  |
| 0.604 | 0.44 (0.029 to 4.186) | 16 (42.1%) | 18 (56.25%) | CC |  |
|  | 1 | 2 (5.26%) | 1 (3.1%) | TT | **Dominant** |
| >0.999 | 0.58 (0.039 to5.215) | 36 (94.73%) | 31 (96.87%) | TC+CC |  |
|  | 1 | 22 (57.89%) | 14 (43.74%) | TT+TC | **Recessive** |
| >0.999 | 1.069 (0.412 to 2.755) | 16 (42.1%) | 18 (56.25%) | CC |  |
|  | 1 | 18 (47.36%) | 19 (59.37%) | TT+CC | **Overdominant** |
| 0.346 | 1.624 (0.645 to 4.325) | 20(53.63%) | 13(40.6%) | TC |  |
|  | 1 | 24 (63.15 %) | 15 (46.87%) | T | **Allelic** |
| 0.543 | 0.726 (0.325 to 1.622) | 36 (94.73%) | 31(96.87%) | C |  |

Values are expressed as number (percentage). ^a^adjusted by age and sex in a logistic regression model. Statistical significance was set at *P*<0.05. CI, confidence interval; KL, Kellgren–Lawrence; OR, odds ratio.
